# Microbial etiologies of ventilator-associated pneumonia (VAP) in intensive care unit of Beni-Suef University’s Hospital

**DOI:** 10.1186/s43088-021-00130-x

**Published:** 2021-07-29

**Authors:** Al Zahraa M. Maebed, Yasser Gaber, Walid Bakeer, Tarek Dishisha

**Affiliations:** 1 Department of Microbiology and Immunology, Faculty of Pharmacy, Nahda University, Beni Suef, Egypt; 2 Department of Microbiology and Immunology, Faculty of Pharmacy, Beni-Suef University, Beni Suef, Egypt; 3 Department of Pharmaceutics and Pharmaceutical Technology, College of Pharmacy, Mutah University, Kerak, 61710 Jordan

**Keywords:** Ventilator-associated pneumonia, VAP, Intensive care unit, Pathogens, *Klebsiella pneumoniae*, *Acinetobacter baumanii*, Antibiotic sensitivity testing

## Abstract

**Background:**

Ventilator-associated pneumonia (VAP) is a major health problem for people intubated in intensive care units (ICUs), leading to increased mortality rates, hospital stay, and treatment costs. In the present study, the core pathogens causing VAP in Beni-Suef University's Hospital, Egypt, was investigated over a study period of 2 years (2017–2019).

**Results:**

Of a total of 213 patients subjected to mechanical ventilation, 60 have developed VAP during their stay in the ICU. The mortality rate reached 41.7% among VAP patients. Sixty bacteria were isolated from an endotracheal aspirate of hospitalized patients. The different isolates were cultured followed by running biochemical tests, sensitivity assays, and automated VITEK®2 System analysis. Unexpectedly, all the isolates were Gram-negative bacteria. *Klebsiella pneumoniae* were the main pathogen encountered (27/60 isolates) followed by *Acientobacter baumannnii* (7/60) and other microorganisms belonging to the genera *Moraxella*, *Escherichia*, and *Pseudomonas* (11/60). Antibiotic sensitivity testing was performed via the VITEK®2 System using up to 16 different antibiotics representing 8 different antibiotic classes and subclasses (aminoglycosides, carbapenems, fluoroquinolones, penicillin/β-lactamase inhibitor, extended-spectrum cephalosporins, aminopenicillins, aminopenicillins/β-lactamase inhibitor, folic acid synthesis inhibitor). Majority of the isolates (28/60) showed a remarkable extensive drug resistance (XDR) pattern, while 15 isolates were multi-drug resistant (MDR) and only 6 were pan-drug resistant (PDR) with regard to antibiotics under evaluation.

**Conclusion:**

The association of VAP with multi-drug-resistant bacteria is alarming, and rapid management is crucial. Identification of core pathogens is essential for identifying the most appropriate technique for infection control.

## Background

Ventilator-associated pneumonia (VAP) is a complication of nosocomial infection that occurs in patients receiving mechanical ventilation for more than 48 h, mainly in intensive care units [1, 2]. It is associated with an increased incidence of morbidity and mortality, long-time admission to hospitals, and increased treatment costs [3]. The mortality rate attributable to VAP was reported as 27% and can reach as high as 43% with antibiotic-resistant organisms [4]. Two different forms of VAP have been reported [5], the early-onset VAP that occurs 48–96 h after intubation and the late-onset VAP that occurs more than 96 h after intubation. The former is associated with antibiotic-susceptible microorganisms including *Staphylococcus aureus*, *Streptococcus pneumoniae*, and *Haemophilus influenzae*, as well as Gram-negative enteric bacilli. While the latter occurs with antibiotic-resistant organisms including methicillin-resistant *S. aureus (MRSA)*, *Pseudomonas aeruginosa*, *Acinetobacter baumannii*, and *Stenotrophomonas maltophilia* [3, 6].

Different reasons stand behind the development of VAP. Initially, the ventilator tracheal tube bypasses the upper airway and facilitates the entry of the bacteria to the lower airway and hence reduces the body’s ability to filter and humidify the air [7]. Additionally, the significant reduction of the cough reflex and impairment of muco-ciliary clearance caused by mucosal injury during intubation increases the chances of microbial infection. The endotracheal tubes also make it easier for the attachment of bacteria to the trachea and hence increases mucus production [8].

The association of VAP with the multi-drug-resistant bacteria in intensive care units (ICUs) has further complicated the situation worldwide [9]. Hence, the development of novel antimicrobials for preventing and/or treating the resulting infections is a necessity. For achieving this, the first step is always the identification of the main causative agents and their resistance pattern. This will facilitate the choice of the proper technique for infection control among the available antimicrobial strategies including antimicrobial peptides (bacteriocins) and enzymes (enzybiotics), predatory bacteria, bacteriophages, and bacteriophage-derived proteins that are considered potential alternatives to conventional antibiotics in the post-antibiotic era, called by the WHO [10, 11].

Therefore, the objective of the current study is to identify the core pathogens causing VAP at Beni-Suef University's (BSU) Hospital which is a public, free hospital operated by the Faculty of Medicine, BSU. The different isolates will be examined for drug resistance pattern against a panel of different antibiotics representing different classes.

## Methods

### Materials and culture media

Blood agar and chocolate agar were procured from HiMedia Laboratories Pvt. Ltd. (Mumbai, India), MacConkey agar from Condalab (Madrid, Spain), and Triple Sugar Iron (TSI) Agar from MAST group Ltd. (Merseyside, UK), and Simmon’s Citrate Agar was obtained from Ltd. (Lancashire, UK). LB agar was prepared in the lab using peptone (Oxford, India), yeast extract (Lab M Limited, UK), NaCl (Biotech, Egypt), and Agar-Agar (B&V Ltd., Italy).

### Study design

This observational, prospective incidence study was undertaken for a 2-year period from mid-2017 to 2019 in the General ICUs of the BSU Hospital, Egypt. Enrolled cases were selected from patients admitted to the emergency ICU with different causes of admission, and the number of days of ventilation and ICU stay was recorded. Only patients that were mechanically ventilated for more than 48 h were considered, excluding the ones having previous evidence of chest infection prior to intubation.

The choice of VAP cases was based on 6 clinical and laboratory criteria: temperature, leukocyte count, volume and purulence of tracheal secretions, oxygen level, chest radiographic findings, and the presence or absence of positive endotracheal aspiration cultures as described earlier for the diagnosis of VAP [12]. Once VAP was clinically diagnosed using clinical or radiological criteria or a combination of both, the specimen was collected by the critical care physician on duty using an aseptic technique. Overall, 60 samples were collected from the endotracheal aspirate.

### Isolation of VAP pathogens

The different isolates were examined microscopically using the Gram-staining technique, then samples were transferred to blood agar, chocolate agar as enriched media, and MacConkey agar as selective and differential media, and the plates were incubated at 37 °C for 48 h.

### Bacterial identification and antibiotic susceptibility testing using the VITEK®2 system

The VITEK®2 compact system (bioMérieux, Marcy l’Etoile, France) at Beni Suef, Egypt, was used for the identification of the different isolates. VITEK®2 compact is an automated microbiology system for the identification of different microorganisms using colorimetric reagent cards. Each card has 64 wells, and each contains a substrate that measures a specific metabolic activity [13]. Sample preparation and operating conditions were according to the manufacturer’s recommendations. The identity of some isolates was further re-confirmed by running a number of biochemical tests including the Triple Sugar Iron (TSI) test and citrate utilization.

The antibiotic susceptibility of the different isolates was determined for 16 different antibiotics belonging to 8 classes and subclasses including aminoglycosides, carbapenems, fluoroquinolones, extended-spectrum cephalosporins, aminopenicillins, aminopenicillins/β-lactamase inhibitors, folic acid synthesis inhibitors, and glycylcyclines. The analyses were performed by the VITEK®2 system using Antibiotic Susceptibility Test (AST) cards, inoculated according to the manufacturer’s guidelines.

## Results

### Antibiotic-resistant VAP in BSU Hospital

The ICU of BSU Hospital has 16 beds. During the study period, 585 patients were admitted to the ICU for different reasons; when cases get worse, they were subjected to mechanical ventilation. Overall, 213 patients were ventilated for more than 5 days, and 60 eligible patients have developed VAP during the study period. Table 1 presents the demographic data (age, sex), cause of entry to the ICU, days of hospital stay, days of mechanical ventilation, and VAP-causing bacteria. Unfortunately, 25 VAP patients died during the 2 years of the study. VAP incidence rate was about 28% (60 patients of a total of 213 patients subjected to mechanical ventilation have developed VAP), and the mortality rate reached 41.6% among VAP cases.

**Table 1:** Demographic data of VAP patients admitted to ICU of BSU Hospital

	Age	Sex	Days of hospital stay	Days of ventilation	Cause of admission	VAP causative bacteria
**1**	63	M	25	10	Liver cell failure	*Klebsiella pneumonia*
**2**	52	M	17	8	Rupture spleen	*Klebsiella pneumonia*
**3**	55	M	20	13	Urinary tract infection (UTI)	*Acinetobacter lwoffii*
**4**	48	M	30	17	Heart disease	*Klebsiella pneumonia*
**5**	33	M	13	5	Trauma	*Klebsiella pneumonia*
**6**	68	M	24	12	Trauma	*Klebsiella pneumonia*
**7**	42	M	10	4	UTI	*Moraxella group*
**8**	70	M	15	8	Diabetic ketoacidosis	*Klebsiella pneumonia*
**9**	38	M	26	14	Liver cell failure	*Klebsiella pneumonia*
**10**	61	M	9	6	Hypovolemic shock	*Pseudomonas aeruginosa*
**11**	46	F	25	14	Cardiogenic shock	*Serratia odorifera*
**12**	31	M	23	9	Trauma	*Klebsiella pneumonia*
**13**	54	F	30	15	Brain disease	*Klebsiella pneumonia*
**14**	28	F	17	7	Trauma	*Klebsiella pneumonia*
**15**	70	M	18	10	Liver cell failure	*Sphinogobacterium thalpophilum*
**16**	65	M	30	14	UTI	*Pseudomonas luteola*
**17**	34	M	14	8	Hyperkalemia	*Moraxella group*
**18**	80	F	28	10	Heart disease	*Acinetobacter baumannii*
**19**	66	M	22	14	UTI	*Escherichia coli*
**20**	34	M	12	5	Trauma	*Klebsiella pneumonia*
**21**	50	M	25	15	Trauma	*Acinetobacter baumannii*
**22**	62	F	20	8	Eclampsia	*Klebsiella pneumonia*
**23**	58	M	28	15	Hypertensive shock	*Klebsiella pneumonia*
**24**	69	M	30	14	Cardiogenic shock	*Acinetobacter lwoffii*
**25**	48	M	28	10	Liver cell failure	*Klebsiella pneumonia*
**26**	60	M	16	7	UTI	*Enterobacter aerogenes*
**27**	72	F	24	10	Diabetic ketoacidosis	*Acinetobacter baumannii*
**28**	68	M	33	17	Brain disease	*Klebsiella pneumonia*
**29**	40	M	10	4	Trauma	*Klebsiella pneumonia*
**30**	29	F	19	6	Eclampsia	*Klebsiella pneumonia*
**31**	64	M	17	10	Heart disease	*Acinetobacter haemolyticus*
**32**	40	M	14	8	Trauma	*Klebsiella pneumonia*
**33**	70	F	20	9	UTI	*Pseudomonas aeruginosa*
**34**	54	M	10	6	Epilepsy	*Acinetobacter baumannii*
**35**	80	M	30	15	Liver cell failure	*Klebsiella pneumonia*
**36**	26	F	13	8	Eclampsia	*Enterobacter aerogenes*
**37**	55	M	20	9	Diabetic ketoacidosis	*Klebsiella pneumonia*
**38**	35	M	10	6	Trauma	*Klebsiella pneumonia*
**39**	44	M	7	5	UTI	*Pseudomonas putida*
**40**	74	M	30	18	Liver cell failure	*Moraxella group*
**41**	67	M	15	8	Kidney disease	*Sphinogobacterium paucimobilis*
**42**	65	M	35	14	Brain disease	*Klebsiella pneumonia*
**43**	71	M	10	4	UTI	*Sphinogobacterium thalpophilum*
**44**	60	M	30	17	Kidney failure	*Acinetobacter baumannii*
**45**	51	M	20	13	Diabetic ketoacidosis	*Klebsiella pneumonia*
**46**	46	M	36	21	Rupture spleen	*Moraxella group*
**47**	57	F	18	7	Diabetic ketoacidosis	*Klebsiella pneumonia*
**48**	35	F	30	10	Eclampsia	*Klebsiella pneumonia*
**49**	78	F	16	9	Liver cell failure	*Pasteurella canis*
**50**	62	F	12	5	Kidney disease	*Klebsiella oxytoca*
**51**	54	F	30	20	Liver cell failure	*Brevundimonas diminyta*/*vesicularis*
**52**	42	F	28	10	Pulmonary disease	*Klebsiella pneumonia*
**53**	49	F	20	8	Rupture spleen	*Pasteurella canis*
**54**	65	F	20	9	Diabetic ketoacidosis	*Pseudomonas luteola*
**55**	80	F	21	7	UTI	*Moraxella group*
**56**	71	F	26	12	Eclampsia	*Enterobacter aerogenes*
**57**	66	F	24	8	Brain disease	*Klebsiella pneumonia*
**58**	57	F	30	16	Diabetic ketoacidosis	*Acinetobacter baumannii*
**59**	56	F	24	13	Cardiogenic shock	*Klebsiella pneumonia*
**60**	43	F	40	17	Trauma	*Acinetobacter baumannii*

Most of the VAP cases were males (*n* = 37; 61.6%) having a mean age of 54.83 ± 8.40 years (range 30–80 years). Trauma was the main cause for ICU admission among VAP male cases, followed by liver cell failure and urinary tract infection (UTI) (Tables 1 and 2). The residual 23 patients were females having a mean age of 23.26 ± 6.52 years (range 25–80 years). Eclampsia and diabetic ketoacidosis were the main causes of ICU admission among female VAP cases (Tables 1 and 2).

**Table 2:** Gender distribution among BSU Hospital VAP cases

Factor		Male	Female
No. of cases		37 (61.6%)	23 (38.3%)
Age range		30–80	25–80
Mean age of the patients		54.83	23.26
SD of age of patient		8.404	6.524
Mean of duration of ventilation/days		10	9
Cause of admission	Liver cell failure	**16.2%**	8.6%
	Rupture spleen	5.4%	4.3%
	Diabetic ketoacidosis	8.1%	**13%**
	Eclampsia	0%	**21.7%**
	Trauma	**18.9%**	8.6%
	Brain disease	5.4%	8.6%
	Kidney disease/failure	8.1%	4.3%
	Cardiogenic shock	2.7%	8.6%
	Heart disease	10.8%	4.3%
	Pulmonary disease	0%	4.3%
	Urinary tract infection	**16.2%**	8.6%
	Hypovolemic shock	2.7%	0%
Died		25 (41.7%)	
Survived		35 (58.3%)	

### Identification and confirmation of bacterial isolates

A total of 60 VAP-associated bacterial pathogens were collected during the study period. All the isolates were obtained from tracheal aspirates. The core pathogens of the VAP were isolated on different culture media: blood agar and chocolate agar as enriched media and MacConkey agar as a selective, differential medium. Most of the isolates appeared as pink colonies on MacConkey agar showing mucoid surface; however, few were black colonies expressing grape-like odor (sweet, fruity smell). The isolates obtained from all the samples were Gram-negative bacteria.

**Fig. 1:**
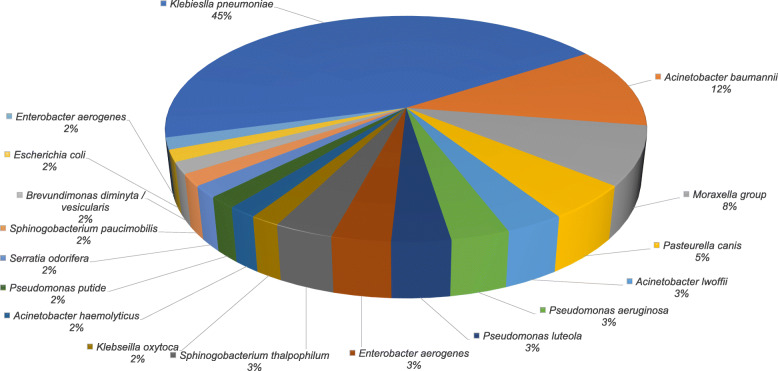
Distribution of infectious agents in patients with ventilator-associated pneumonia in the ICU of BSU Hospital

The identification of the isolates was done by the VITEK®2 system. Figure 1 presents the distribution of the infectious agents among VAP cases. *Klebsiella* species (*n* = 28; 46.7%) were the main responsible for VAP; 27 were identified as *K. pneumoniae* while 1 was *K. oxytoca*. *Acinetobacter* species (*n* = 10; 16.7%) came second including *A. baumannii* (7 cases), *A. lwoffii* (2 cases), and *A. haemolyticus* (1 case). The remaining cases were as follows: *Moraxella* group (5), *Pseudomonas aeruginosa* (2), *Pseudomonas luteola* (2), *Pseudomonas putida* (1), *Serratia odorifera* (1), *Sphinogobacterium thalpophilum* (2), *Sphinogobacterium paucimobilis* (1), *Brevundimonas diminyta*/*vesicularis* (1), *Escherichia coli* (1), and *Enterobacter aerogenes* (1).

A number of biochemical tests were conducted for confirming the identity of some isolates using the TSI test which gave acid slant and acid butt accompanied with gas production with *K. pneumoniae* while was negative with *Acinetobacter* being a non-fermentative microorganism. On the other hand, *E. coli* had similar results to *K. pneumoniae* accompanied with more gas production, however was negative to citrate test [14].

### Antibiotic susceptibility testing of VAP isolates

Isolates were tested for antimicrobial susceptibility using the VITEK®2 system and partly using the modified Kirby-Bauer disc diffusion method [15]. The sensitivity of the different isolates was recorded as sensitive, medium, or resistant. Based on this evaluation, the isolates were categorized as follows: multi-drug resistance (MDR) was designated for isolates non-susceptible to at least one agent in at least three different antimicrobial categories, extensive drug resistance (XDR) for isolates non-susceptible to at least one agent in all but at maximum 2 categories, and pan-drug resistance (PDR) was used for isolates non-susceptible to all listed antimicrobial agents. This categorization was employed according to the classification made by the European Centre for Disease Prevention and Control (ECDC) and the Centre for Disease Control and Prevention (CDC) and other expert opinions [16, 17].

The antimicrobial susceptibility of *K. pneumoniae* isolates was tested against 8 different classes of antibiotics/antibiotic combinations. These included aminoglycosides (gentamicin, tobramycin, amikacin), carbapenems (meropenem, imipenem), fluoroquinolones (levofloxacine, ciprofloxacin), penicillin/β-lactamase inhibitor combination (piperacillin/tazobactam), extended-spectrum cephalosporins (cefepime, cefazolin, cefoxitin, ceftazidime, ceftriaxone), aminopenicillins (ampicillin), aminopenicillins/β-lactamase inhibitor combination (ampicillin/sulbactam), and folic acid synthesis inhibitor (trimethoprim/sulfamethoxazole). The percentage antimicrobial resistance of *K. pneumoniae* isolates ranged between 67 and 96% towards tested antibiotics. Most of the isolates showed multi-drug and extensive drug resistance patterns except for only one isolate which showed a pan-resistance pattern (considering only the examined groups) (Table 3). The isolates were more sensitive to aminoglycoside, fluoroquinolones, and aminopenicillins/β-lactamase inhibitor combination.

**Table 3:**
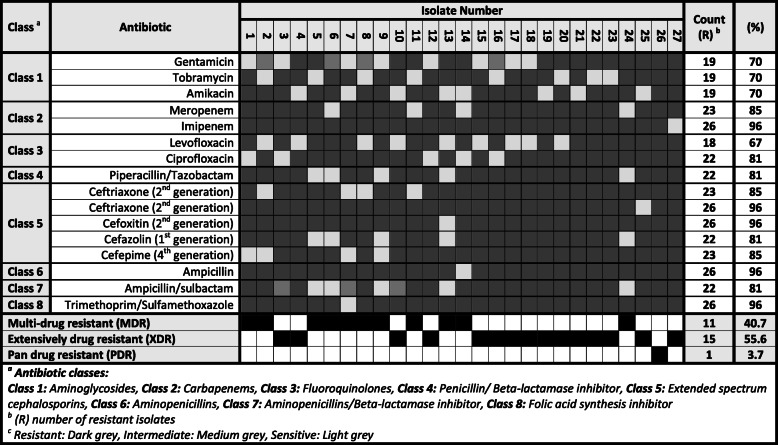
Antibiotic resistance pattern among the different *Klebsiella* isolates

Resistant, dark gray; intermediate, medium gray; sensitive, light gray

^a^Antibiotic classes: class 1, aminoglycosides; class 2, carbapenems; class 3, fluoroquinolones; class 4, penicillin/beta-lactamase inhibitor; class 5, extended-spectrum cephalosporins; class 6, aminopenicillins; class 7, aminopenicillins/beta-lactamase inhibitor; and class 8, folic acid synthesis inhibitor

^b^*R* number of resistant isolates

On the other hand, the *Acinetobacter* isolates had a remarkable resistance with most of them showing pan- and extensively drug resistance patterns (Table 4). *Acinetobacter* spp. had about 71–100% resistance rates to all tested antimicrobials, including carbapenems (100%), extended-spectrum cephalosporins (86–100%), fluoroquinolones (100%), aminoglycosides (86–100%), folic acid synthesis inhibitor (86%), glycylcyclines (100%), and aminopenicillins/beta-lactamase inhibitor (71%) (Table 4).

**Table 4:**
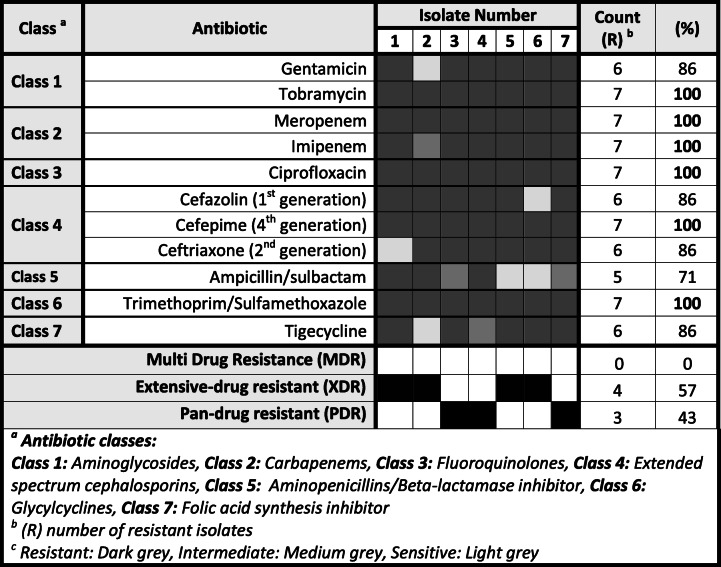
Antibiotic resistance pattern among the different *Acinetobacter* isolates

Resistant, dark gray; intermediate, medium gray; sensitive, light gray

^a^Antibiotic classes: class 1, aminoglycosides; class 2, carbapenems; class 3, fluoroquinolones; class 4, extended-spectrum cephalosporins; class 5, aminopenicillins/beta-lactamase inhibitor; class 6, glycylcyclines; and class 7, folic acid synthesis inhibitor

^b^*R* number of resistant isolates

Table 5 presents the resistance pattern of all the remaining isolates which showed different degrees of resistance. Sample numbers 3 and 14 representing *Pseudomonas luteola* were shown as pan-drug resistant; however, all the remaining samples vary between multi- and extensive drug-resistant (XDR).

**Table 5:**
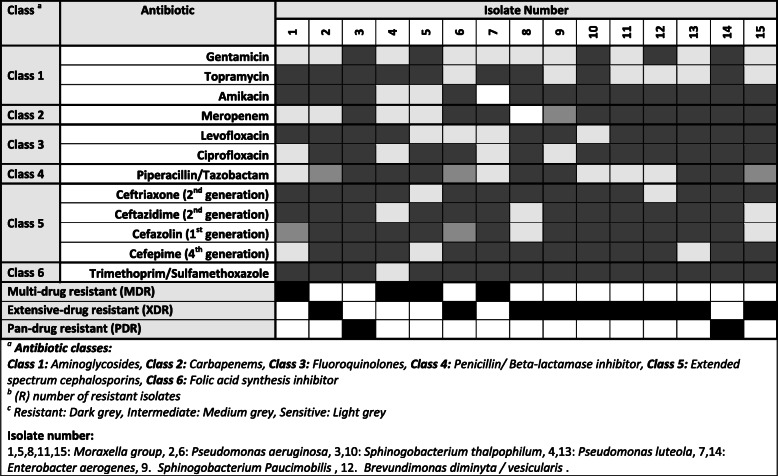
Antibiotic resistance pattern among the different other different isolates

Resistant, dark gray; intermediate, medium gray; sensitive, light gray

Isolate number: 1, 5, 8, 11, and 15—*Moraxella* group; 2 and 6—*Pseudomonas aeruginosa*; 3 and 10—*Sphinogobacterium thalpophilum*; 4 and 13—*Pseudomonas luteola*; 7 and 14—*Enterobacter aerogenes*; 9—*Sphinogobacterium Paucimobilis*; 12—*Brevundimonas diminyta*/*vesicularis*

*R* number of resistant isolates

^a^Antibiotic classes: class 1, aminoglycosides; class 2, carbapenems; class 3, fluoroquinolones; class 4, penicillin/beta-lactamase inhibitor; class 5, extended-spectrum cephalosporins; and class 6, folic acid synthesis inhibitor

## Discussion

Oxygen which our cells need to operate comes from the outside environment and is exchanged with carbon dioxide in our respiratory system. The lungs are the place where gas exchange takes place. The lungs are especially helpless to disease since they are associated with the exterior environment that contains irresistible or poisonous species. When the lung alveoli are inflamed or infected, pneumonia occurs, which is known as the “captain of the men of death” as described by Dr. William Osler, the founder of modern medicine [18].

Despite extensive strategies for managing and decreasing VAP incidence, it remains to be the main cause of most deaths in patients with nosocomial infections [19]. This is in agreement with other studies reporting that lower respiratory tract infection is the major cause of death among different nosocomial infections [20, 21]. The overall incidence rate of VAP in ICUs ranged from 10 to 70% as reported in a review article by Krishnamurthy et al. [22], indicating that different factors might contribute to the development of VAP. In the present study, the incidence rate of VAP was around 28% (60 patients of a total of 213 patients).

Different factors were reported to increase the probability of VAP. Intubation is considered the most common cause for developing nosocomial pneumonia [23], besides the aspiration of infected secretions from the oropharynx [24]. In the present study, most VAP cases were males probably due to smoking habits which is culturally predominant between males than females. This comes in agreement with previous studies which reported that smoker patients and those with underlying lung diseases, including chronic obstructive pulmonary disease (COPD), cystic fibrosis, congestive heart failure, and lung cancer, are more vulnerable to VAP owing to abnormalities in the lung structure and function [25].

The organisms causing VAP vary according to case mix, prior antibiotic exposure, the length of stay in the ICU, length of mechanical ventilation, patient characteristics, clinical circumstances, and geographic location (even between units in the same hospital) which emphasize the need for local epidemiological and microbiological evaluation [26]. Regarding the results of the present study, the most common microorganisms isolated from VAP cases were Gram-negative bacilli. Surprisingly, no infections were recorded based on Gram-positive isolates, indicating that probably, the antibiotic regime used as a rule of thumb in the ICU and the cleaning protocols diminishes the possibility of their growth. Similar results were recently reported in the pediatric intensive care unit of King Abdulaziz Medical City, Saudi Arabia, during the evaluation of the VAP prevention bundle [27]. The prevention bundle included elevating the bed’s head to avoid aspiration of oropharynx secretions, providing mouth hygiene to reduce oropharynx colonization, keeping ventilator circuits clean and dry to reduce device contamination, hand washing before and after touching the patients, using a cuffed endotracheal tube to avoid aspiration of oropharynx secretions, sedation holiday for deeply sedated patients every morning, and using anti-reflex prophylaxis. This resulted in the omission of Gram-positive bacteria-mediated VAP [27]. Other studies at Egyptian Nasser Institute’s ICU [28], Saudi Arabian King Abdul-Aziz Medical City in Riyadh [29], and ICUs of different hospitals in an urban town in India [22] showed a higher rate of Gram-negative bacteria than Gram-positive ones.

Among the isolated Gram-negative bacilli, *Klebsiella* were the most common, followed by *Acinetobacter* and *Pseudomonas*. This agrees with a previous study conducted by Kanafani et al. [30] who have reported similar results for the VAP pathogen. *Klebsiella* spp., especially *Klebsiella pneumoniae*, are among the most common opportunistic nosocomial pathogens, ranked third with regard to drug-resistant infections in hospitals [31]. The digestive tract of ICU patients, the hands of the nursing staff, and the unclean endotracheal tube are considered the most significant reservoir of VAP pathogens [32].

On the other hand, core pathogens of VAP were different in several studies. *K. pneumoniae* was responsible for only a small fraction of VAP infections among ICU patients in Pakistani Clinic representing only 6.6%, while MRSA was the main pathogen (40% of VAP cases) [33]. In a different study conducted to correlate the VAP cases and causative organisms, Rello (2004) [34] reported that *H. influenza* and *M. catarrhalis* were the main findings in COPD patients, and *P. aeruginosa* and *S. aureus* were predominant in patients with cystic fibrosis, while *S. aureus* was the core pathogen in the case of trauma and neurologic patients*. Acinetobacter* also remains to be one of the core pathogens causing deadly VAP. It was responsible for most of the VAP cases in King Abdelaziz Medical City (KAMC) in Riyadh, Saudi Arabia [35].

Moreover, the antibiotic resistance aggravates the situation. The high rates of microbial resistance reported in the different studies are terrifying. *Klebsiella pneumoniae*, the causative agent of VAP in this study, was resistant to multiple antibiotics the same as the results of the study of Hudson et al. [36]. *Klebsiella* species with the ability to produce extended-spectrum β-lactamases (ESBL) are resistant to virtually all β-lactam antibiotics, except carbapenems. Other frequent resistance targets include aminoglycosides, fluoroquinolones, tetracyclines, chloramphenicol, and trimethoprim/sulfamethoxazole also similar to the study of Nathisuwan et al. [37].

A study conducted to evaluate the device-associated infection at Cairo University Hospital reported that around 70% of tested *E. coli* and *K. pneumoniae* isolates produced extended-spectrum β-lactamases [38]. In the USA, around 20% of *E. coli* and *K. pneumoniae* VAP isolates had extended-spectrum cephalosporin resistance. Resistance rates for other organisms are also substantially higher in Egypt. For instance, 100% of *Acinetobacter* spp*.* isolates from hospital-acquired infections in Egypt are MDR versus approximately 70% of isolates in the NHSN; 93% of *S. aureus* isolates tested in Egypt were methicillin-resistant compared with 50% in the NHSN [39].

## Conclusions

VAP is an extreme infection that has to be managed as early as possible. The association of VAP with the multi-, extensive-, and pan-drug-resistant bacteria specially for patients admitted to the ICUs is alarming and resulting in a considerable medical problem worldwide. As a result, the World Health Organization (WHO) has released an alarming infographic in 2014 motivating the researchers and companies to develop novel antimicrobials preparing for the post-antibiotic era. Different strategies are under investigation worldwide including bacteriophages, phage-derived proteins, bacteriocins, and metabolomics-based antibiotic mining, among others.

## Data Availability

The datasets used and/or analyzed during the current study are available from the corresponding author on reasonable request.

## References

[1] Kollef MH (1999). The prevention of ventilator-associated pneumonia. N Engl J Med.

[2] Zack JE, Garrison T, Trovillion E, Clinkscale D, Coopersmith CM, Fraser VJ, Kollef MH (2002). Effect of an education program aimed at reducing the occurrence of ventilator-associated pneumonia. Crit Care Med.

[3] Trouillet J-L, Chastre J, Vuagnat A, Joly-Guillou M-L, Combaux D, Dombret M-C, Gibert C (1998). Ventilator-associated pneumonia caused by potentially drug-resistant bacteria. Am J Respir Crit Care Med.

[4] Craven DE (2000). Epidemiology of ventilator-associated pneumonia. Chest.

[5] Rello J, Torres A (1996). Microbial causes of ventilator-associated pneumonia. Seminars in respiratory infections.

[6] American_Thoracic_Society (1996). Hospital-acquired pneumonia in adults: diagnosis, assessment of severity, initial antimicrobial therapy, and preventive strategies. A consensus statement, American Thoracic Society, November 1995. Am J Respir Crit Care Med.

[7] Morehead RS, Pinto SJ (2000). Ventilator-associated pneumonia. Arch Intern Med.

[8] De Rosa FG (2003). Ventilator-associated pneumonia: current management strategies. Inf Med.

[9] Ryan JF, Newman A, Jacobs M (2000). The pharmaceutical century: ten decades of drug discovery.

[10] Nicastro J, Wong S, Khazaei Z, Lam P, Blay J, Slavcev RA (2016) Bacteriophage applications-historical perspective and future potential. Springer International Publishing (Cham, Switzerland). 10.1007/978-3-319-45791-8

[11] Reuter M, Kruger DH (2020). Approaches to optimize therapeutic bacteriophage and bacteriophage-derived products to combat bacterial infections. Virus Genes.

[12] Horan TC, Andrus M, Dudeck MA (2008). CDC/NHSN surveillance definition of health care–associated infection and criteria for specific types of infections in the acute care setting. Am J Infect Control.

[13] Pincus DH (2006). Microbial identification using the bioMérieux Vitek®2 system. Encyclopedia of Rapid Microbiological Methods.

[14] Hogan J, Smith KL (2003). Coliform mastitis. Vet Res.

[15] Wayne PA (2011). Clinical and laboratory standards institute. Performance standards for antimicrobial susceptibility testing.

[16] Ortmann AC, Suttle CA (2009) Determination of virus abundance by epifluorescence microscopy. In: Methods in Molecular Biology (501). Springer (Clifton, N.J.), pp 87–95. 10.1007/978-1-60327-164-6_10 10.1007/978-1-60327-164-6_10 19066814

[17] Tapsall JW, Ndowa F, Lewis DA, Unemo M (2009). Meeting the public health challenge of multidrug-and extensively drug-resistant *Neisseria gonorrhoeae*. Expert Rev Anti Infect Ther.

[18] Osler W (1892). The principles and practice of medicine.

[19] Kollef MH (2005). What is ventilator-associated pneumonia and why is it important?. Respir Care.

[20] Celis R, Torres A, Gatell JM, Almela M, Rodríguez-Roisin R (1988). Nosocomial pneumonia. A multivariate analysis of risk and prognosis. Chest.

[21] Gross PA, Neu HC, Aswapokee P, Van Antwerpen C, Aswapokee N (1980). Deaths from nosocomial infections: experience in a university hospital and a community hospital. Am J Med.

[22] Krishnamurthy V, Vijay Kumar GS, Prashanth HV, Prakash R, Kumar MS (2013). Ventilator associated pneumonia: bacterial isolates and its antibiotic resistance pattern. Int J Biol Med Res.

[23] Rello J, Diaz E (2003). Pneumonia in the intensive care unit. Crit Care Med.

[24] Estes RJ, Meduri GU (1995). The pathogenesis of ventilator-associated pneumonia. Care Med.

[25] Hunter JD (2006). Ventilator associated pneumonia. Postgrad Med J.

[26] Park DR (2005). The microbiology of ventilator-associated pneumonia. Respir Care.

[27] Osman S, Al Talhi YM, AlDabbagh M, Baksh M, Osman M, Azzam M (2020). The incidence of ventilator-associated pneumonia (VAP) in a tertiary-care center: comparison between pre- and post-VAP prevention bundle. J Infect Public Health.

[28] Abdel-Latif W (2013). The impact of improvement project on ventilator associated pneumonia incidence rate at Nasser Institute intensive care unit in Cairo. Egypt J Med Lab Sci.

[29] Al-Dorzi HM, El-Saed A, Rishu AH, Balkhy HH (2012). The results of a 6-year epidemiologic surveillance for ventilator-associated pneumonia at a tertiary care intensive care unit in Saudi Arabia. Am J Infect Control.

[30] Kanafani Z, Kara L, Hayek S, Kanj S (2003). Ventilator-associated pneumonia at a tertiary-care center in a developing country: incidence, microbiology, and susceptibility patterns of isolated microorganisms. Infect Control Hosp Epidemiol.

[31] Pendleton JN, Gorman SP, Gilmore BF (2013). Clinical relevance of the ESKAPE pathogens. Expert Rev Anti Infect Ther.

[32] Coovadia YM, Johnson AP, Bhana RH, Hutchinson GR, George RC, Hafferjee IE (1992). Multiresistant *Klebsiella pneumoniae* in a neonatal nursery: the importance of maintenance of infection control policies and procedures in the prevention of outbreaks. J Hosp Infect.

[33] Ahmad S, Bacha N, Bakht J, Jawad A (2017). Characterization of pathogens involved in ventilator associated pneumonia in surgical and medical intensive care units: a single center experience. Pak J Pharm Sci.

[34] Rello J (2004). Bench-to-bedside review: therapeutic options and issues in the management of ventilator-associated bacterial pneumonia. Crit Care.

[35] Balkhy HH, El-Saed A, Maghraby R, Al-Dorzi HM, Khan R, Rishu AH, Arabi YM (2014). Drug-resistant ventilator associated pneumonia in a tertiary care hospital in Saudi Arabia. Ann Thorac Med.

[36] Hudson CM, Bent ZW, Meagher RJ, Williams KP (2014). Resistance determinants and mobile genetic elements of an NDM-1-encoding Klebsiella pneumoniae strain. PLoS One.

[37] Nathisuwan S, Burgess DS, Lewis J (2001). Extended-spectrum β-lactamases: epidemiology, detection, and treatment. Pharmacotherapy.

[38] El-Kholy A, Saied T, Gaber M, Younan MA, Haleim MM, El-Sayed H (2012). Device-associated nosocomial infection rates in intensive care units at Cairo University Hospitals: first step toward initiating surveillance programs in a resource-limited country. Am J Infect Control.

[39] Sievert DM, Ricks P, Edwards JR, Schneider A, Patel J, Srinivasan A, Kallen A, Limbago B, Fridkin S (2013). Antimicrobial-resistant pathogens associated with healthcare-associated infections: summary of data reported to the National Healthcare Safety Network at the Centers for Disease Control and Prevention. Infect Control Hosp Epidemiol.

